# WORKSHOP PANEL REPORT ON EVIDENCE GAPS AND RESEARCH OPPORTUNITIES

**DOI:** 10.1093/geroni/igz038.2746

**Published:** 2019-11-08

**Authors:** Heather Allore

**Affiliations:** Yale University, New Haven, Connecticut, United States

## Abstract

Although varied in treatment regimens and outcomes assessment, trials show safe and effective osteoporosis drug therapies up to 5 years in reducing the incidence of vertebral fractures. Trials provide evidence mainly for white postmenopausal women, while other populations (e.g., men, spectrum of race and ethnicities, residents in facilities, and people with advanced and multiple comorbid conditions) were absent or underrepresented. Evidence for osteoporosis drug therapies is lacking for people who meet neither bone mineral density nor fracture criteria for osteoporosis but are at elevated risk due to other health, genetic, or medication use factors. Fewer studies have shown that some osteoporosis drug therapies reduce the incidence of non-vertebral, including hip fractures. Notably, the workshop participants emphasized the importance of fracture on patient morbidity and survival; however, the trial results presented no data on non-fracture patient outcomes or sequelae of fractures, such as functional status, mobility, hospitalizations, and nursing home placement.

